# Provoking factors for postpartum chronic hypertension in women with preceding gestational hypertension/preeclampsia: A longitudinal cohort study of 22,798 pregnancies

**DOI:** 10.7150/ijms.39432

**Published:** 2020-02-10

**Authors:** Kuo-Hu Chen, Li-Ru Chen

**Affiliations:** 1 Department of Obstetrics and Gynecology, Taipei Tzu-Chi Hospital, The Buddhist Tzu-Chi Medical Foundation, Taipei, Taiwan; 2 School of Medicine, Tzu-Chi University, Hualien, Taiwan; 3 Department of Physical Medicine and Rehabilitation, Mackay Memorial Hospital, Taipei, Taiwan; 4 Department of Mechanical Engineering, National Chiao-Tung University, Hsinchu, Taiwan

**Keywords:** gestational hypertension, preeclampsia, chronic hypertension, pregnant weight gain, gestational diabetes mellitus

## Abstract

**Background**: A proportion of women with pregnancies complicated by gestational hypertension/preeclampsia (GH-PE) will have persistent postpartum chronic hypertension (CHTN). Common risk factors for postpartum CHTN include older age, pre-existing CHTN, smoking, pre-pregnancy obesity (elevated BMI), and co-morbidities such as thyroid disorders. However, most of explored risk factors are pre-pregnancy factors, and were mainly based on studies with small sample size.

**Methods**: To investigate provoking pre-pregnancy and intra-pregnancy factors for postpartum CHTN in women with preceding GH-PE, the cohort study enrolled 22,798 index pregnancies to analyze individual characteristics, co-morbidities and postpartum outcomes after excluding women with pre-existing CHTN.

**Results**: Among 2,132 GH-PE pregnancies, 428 (20.1%) were complicated with postpartum CHTN. After adjustment, logistic regression analysis revealed excessive pregnant weight gain (≥10 kgw at 28 weeks' gestation) (OR: 14.50, 95% CI: 11.02-19.08) and gestational diabetes mellitus (GDM) (OR: 6.25, 95% CI: 4.98-7.85) were major risk factors for developing CHTN, other than age (OR: 1.80, 95% CI: 1.68-1.93), pre-pregnancy BMI (OR: 3.15, 95% CI: 2.75-3.60), severity of GH-PE (OR: 2.46, 95% CI: 1.97-3.07), smoking (OR: 1.79, 95% CI: 1.35-2.38), and overt DM (OR: 2.30, 95% CI: 1.73-3.06).

**Conclusion**: Excessive pregnant weight gain and GDM are major intra-pregnancy risk factors for postpartum CHTN in women with preceding GH-PE. Future studies should investigate interventions such as a healthy diet, appropriate physical exercise and avoidance of excessive pregnant weight gain as a means to reduce the frequency of CHTN following pregnancy.

## Introduction

In the United States, gestational hypertension/preeclampsia (GH-PE) is the most common medical complication during pregnancy, with an overall prevalence of 6%-8% 1. PE is one of the main causes of maternal and perinatal morbidity and mortality, especially in low- and middle-income countries 2-4, and GH-PE predisposes mothers and fetuses to cardiovascular disease later in life 3, 5-8. The etiology of PE is unclear. However, a two-stage model of abnormal trophoblastic invasion during placental implantation, leading to placenta ischemia and insufficiency, with subsequent endothelial injury, is believed to underlie the pathophysiology of GH-PE 1, 2, 9-11. In consequence, endothelial cells may be injured by a combination of factors induced by placental hypoxia and an abnormal maternal inflammatory response 12. Endothelial dysfunction leads to abnormal vasoconstriction, increased vascular permeability, and thrombosis, which induce systemic hypertension, edema, and systemic and placental infarcts, respectively 12. Elevated BP may be related to decreased production of nitrogen oxide (NO) 2, leading to endothelial dysfunction and decreased vasodilatatory responses. Chronic inflammation, oxidative stress, insulin resistance and endothelial cell activation related to pregnancy have been described in obese, diabetic or pregnant women, and have been associated with hypertension in both pregnant and non-pregnant women 10, 12, 13-17. During pregnancy, abnormal morphological and blood-flow changes of placenta, including preterm grade III placental calcification (diffuse echogenic lines or indentations extending from the chorionic plate to the basal layer of the placenta before 37 weeks' gestation) and high impedance of uterine arteries (pulsatility index > 1.2 or the finding of a diastolic notch under uterine artery Doppler velocimetry), may be an implication of poor placental function and perinatal outcomes, and can serve as a marker of further intrapartum and postpartum events 3, 5, 9, 11, 17-18. Using ultrasonography, we have previously shown that preterm grade III placental calcification is a major risk factor for progression of GH to PE 17, adverse maternal and neonatal outcomes 18, 19, and even stillbirth 20.

Some women with pregnancies complicated by GH-PE will have persistent postpartum chronic hypertension (CHTN) 8, 21. Previous studies have revealed that older age, pre-existing chronic hypertension, smoking, pre-pregnancy obesity (elevated body mass index [BMI]), and co-morbidities such as thyroid disorders are common risk factors for postpartum CHTN 22, 23. Obesity 10, 12, 16 and excessive weight gain 17 are risk factors for elevated BP, resulting from chronic inflammation, oxidative stress, and placental endothelial cell activation 10, 12, 16. However, most of explored risk factors for CHTN are pre-pregnancy factors, based on studies with small sample sizes (< 2,000 GH-PE women) 22, 23. Thus, by analyzing a large population (>20,000 index pregnancies), the current study aimed to identify both pre-pregnancy and intra-pregnancy provoking factors for postpartum CHTN, in women with preceding GH-PE.

## Materials and methods

This longitudinal cohort study was conducted in a tertiary teaching hospital with an average of ≥ 200 deliveries per month. The study was approved by the Institutional Review Board of Taipei Tzu-Chi Hospital, Taiwan, followed the principle of the declaration of Helsinki, and met the guidelines of the responsible governmental agency.

During the period between 1, July, 2005 and 30, June, 2017, all eligible pregnancies of women in the obstetric clinics were enrolled for the study to decrease selection bias. Initial screening was performed to exclude pregnancies in women who had missing medical data, or did not deliver at our hospital. Patients were also excluded in case of inconsistent description for disease diagnosis and progression, and wrong or inappropriate coding of diagnoses and treatment. Because the current study focused on provoking factors for postpartum CHTN in GH-PE pregnancies, women who had pre-existing chronic hypertension were excluded from the study. The diagnosis of GH is made by ≥ 2 measurements of hypertension (systolic BP ≥ 140 mmHg or diastolic BP ≥ 90 mmHg) noted at 20, 24, and 28 weeks gestation without proteinuria and not treated using anti-hypertensive medications, including hydralazine, methdopa, and nifedipine. According to *Williams Obstetrics*, PE is defined as hypertension during pregnancy (BP ≥ 140/90 mmHg) accompanied by proteinuria (≥ 300 mg /24 h or ≥ 1+ on dipstick) 2. The severity of GH-PE is classified by the occurrence of PE or not (GH alone).

Basic information including age, pre-pregnancy BMI, parity, smoking, general medical history and co-morbidities were retrieved at the first antepartum visit. Identification of multi-fetal pregnancy, diagnoses of pre-existing chronic hypertension, GH-PE and gestational diabetes mellitus (GDM) were made on subsequent antepartum visits. The diagnosis of persistent postpartum CHTN is confirmed by records of hypertension noted at both one month and one year after delivery. Excessive pregnant weight gain was defined as ≥ 10 kgw of weight gain at 28 weeks when compared to 8 weeks, according to the recommendations for weight gain during pregnancy 2.

Using SPSS 19.0 (SPSS, Inc., Chicago, IL, USA), descriptive statistics, chi-square (χ^2^) test, and Student t-test, were performed as appropriate to compare the characteristics and outcomes of pregnancies. Logistic regression analysis using stepwise selection method was performed to explore the provoking factors for postpartum CHTN by reporting odds ratios (ORs) and 95% confidence intervals (CIs) after adjusting for maternal age, pre-pregnancy BMI, parity, multi-fetal pregnancy, excessive pregnant weight gain, marital status, smoking, delivery type, severity of GH-PE and co-morbidities.

## Results

**Figure 1:**
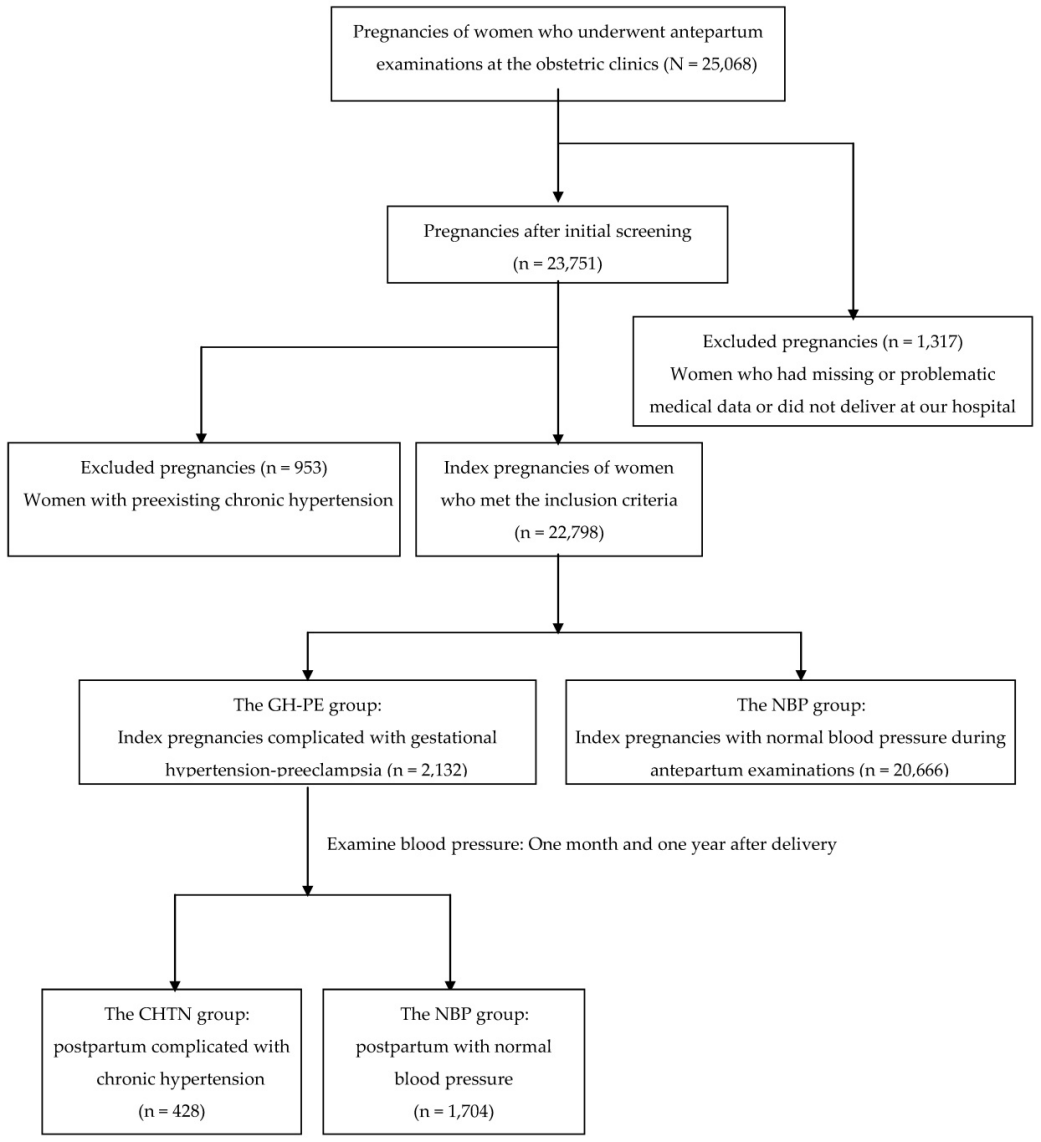
A flow diagram for pregnancies classified according to blood pressure.

A flow diagram for pregnancies classified according to blood pressure is shown in Figure 1. There were 25,068 pregnancies of women who underwent antepartum examinations at the obstetric clinics. After initial screening excluded women who had missing data or did not deliver at our hospital (n = 1,317), 23,751 pregnancies were eligible for further analysis. A further 953 index pregnancies were excluded due to preexisting chronic hypertension, with 22,798 index pregnancies meeting the inclusion criteria. Of the 22,798 index pregnancies 2,132 were complicated by elevated BP (the GH-PE group) and the other 20,666 were presented with normal BP during antepartum examinations. Among the postpartum women in the GH-PE group, 428 (20.1%) were complicated by chronic hypertension (the CHTN group), and 1,704 (79.9%) had normal blood pressure (the NBP group).

The characteristics and co-morbidities of the GH-PE pregnancies between the postpartum CHTN and NBP groups are compared in Table 1. There were significant differences in maternal age, pre-pregnancy BMI, the ratios of excessive pregnant weight gain, severity of GH-PE, overt DM and GDM (all *p* < 0.001), and smoking (*p* < 0.05) between the two groups. Logistic regression analysis was used to explore provoking factors for the occurrence of postpartum CHTN in GH-PE women. After adjustment, older maternal age (OR=1.80; 95% CI=1.68-1.93), elevated pre-pregnancy BMI (OR=3.15; 95% CI=2.75-3.60), excessive pregnant weight gain (OR=14.50; 95% CI=11.02-19.08), cigarette smoking (OR=1.79; 95% CI=1.35-2.38), severity of GH-PE (OR=2.46; 95% CI=1.97-3.07), overt DM (OR=2.30; 95% CI=1.73-3.06) and GDM (OR=6.25; 95% CI=4.98-7.85) were associated with an increased risk of postpartum CHTN. Remarkably, excessive pregnant weight gain and GDM were two major provoking factors for postpartum CHTN.

## Discussion

By excluding women with pre-existing chronic hypertension, this current study focused mainly on the effect of pregnancy on subsequent postpartum CHTN. Compared to the incidence (821/20,666 = 3.97%) of postpartum CHTN in normotensive pregnancies, women which developed hypertension during pregnancy (GH-PE) had a near five-fold incidence (20.1%) of postpartum CHTN. Moreover, our results revealed that intra-pregnancy factors (excessive pregnant weight gain, GDM and severity of GH-PE) had greater effects on postpartum CHTN, compared with pre-pregnancy factors (pre-pregnancy BMI, smoking, pre-existing overt DM and other co-morbidities). Thus women with excessive pregnant weight gain or GDM should be screened for hypertension after delivery, in order to allow for prompt management.

The result of the current study revealed that both excessive pregnant weight gain and pre- pregnancy obesity are risk factors for postpartum CHTN. The mechanisms of obesity-associated HTN are complex. Obesity and excessive deposition of adipose tissues may alter endothelial function and enhance BP via chronic inflammation, oxidative stress, sodium retention, insulin resistance and placental endothelial cell activation 10, 12-17, 24. Adipose tissue of obese patients becomes resistant to insulin and is the site of altered secretion of molecules such as adiponectin, leptin, resistin, tumor necrosis factor (TNFα) and IL-6, which induce obesity-associated cardiovascular disease 13, 14. During the early phases of obesity, increased renal tubular reabsorption leads to primary sodium retention. Extracellular-fluid volume is expanded and the kidney-fluid apparatus is resetted to a hypertensive level, consistent with a volume overload-hypertension model 14, 15. Plasma renin activity, angiotensinogen, angiotensin II (AngII) and aldosterone values display significant increase during obesity and DM 14, 15. Obesity-associated microvascular dysfunction and resultant HTN is thought to arise via two signaling pathways. In the endothelial cells, insulin-mediated PI3-kinase (PI3K)/NO activation leads to vasodilation. In contrast, endothelin 1 (ET-1) production activates the MAPK pathway, leading to vasoconstriction 14, 25. The mediators AngII, TNFα, and free fatty acids (FFA), which are released from the adipose tissues of obese patients, can inhibit the PI3-kinase (PI3K) pathway and stimulate the MAPK pathway 25. In pregnant women with excessive weight gain, increased sodium retention, and metabolic disturbances such as enhanced oxidative stress and insulin resistance, may underlie their predisposition to develop postpartum CHTN 10, 12-14, 16, 25.

GDM was also found to be a significant predictor for postpartum CHTN in the current study. Elevated BP in GDM patients appears to be closely related to increased circulatory fluid volume and increased peripheral vascular resistance 15, 26. Specifically, pregnant patients with diabetes mellitus (including overt DM and GDM) experience increased peripheral artery resistance and increased body fluid volume, secondary to hyperinsulinemia and hyperglycemia. Both of these mechanisms elevate systemic BP 15, 26.

In comparison to GH alone, the occurrence of PE is associated with more profuse systemic impairment, including vascular endothelial injury to the cardiovascular system 1-3, 27. Our result is similar to another research reporting increased risk of subsequent hypertension among pregnant women with more severe PE 27. In addition, cigarette smoking (OR=1.79) is a provoking factor for postpartum CHTN, and the finding is consistent with our previous report which demonstrated that cigarette smoking slightly increased the risk of GH and PE (OR=1.42~1.68) 17. It is well known that cigarette smoking can result in generalized endothelial dysfunction. Hence, cigarette smoking cessation is helpful to prevent both GH-PE and subsequent CHTN 17.

Compared with previous reports that focused mainly on pre-pregnancy factors, the current report is the first study to investigate both pre-pregnancy and intra-pregnancy factors, using a large cohort (>20,000 index pregnancies). Importantly, our results suggest modifiable risk factors such as excessive pregnant weight gain and GDM are associated with increased risk for postpartum CHTN. The limitation of our study arises from some characteristics such as race and socio-economic status, which were not considered and could affect the results. Currently, there are still many potential factors for predisposing to persistent postpartum CHTN, which remain unexplored and warrant further investigation.

## Conclusion

Excessive pregnant weight gain and GDM are major intra-pregnancy risk factors for postpartum CHTN in women with preceding GH-PE. Women with excessive pregnant weight gain or GDM should be screened for hypertension after delivery. Future studies should investigate interventions such as a healthy diet, appropriate physical exercise and avoidance of excessive pregnant weight gain as a means to reduce the frequency of CHTN following pregnancy.

## Figures and Tables

**Table 1:** Comparison of GH-PE pregnancies between persistent postpartum chronic hypertension (CHTN) and normal blood pressure (NBP) groups

	CHTN group (n = 428)		NBP group (n = 1704)		Statistics		
	N	%	N	%	*p*-value	OR^a^	95% CI
	(mean ± SD)		(mean ± SD)				
Age (years)	32.72 ± 1.21		29.95 ± 3.21		<0.001***	1.80***	1.68-1.93
Pre-pregnancy BMI (kg/m^2^)	23.51 ± 1.09		22.48 ± 1.24		<0.001***	3.15***	2.75-3.60
Parity					0.619		
0	27	63.8	1084	63.6		reference	
1	119	27.8	452	26.5		1.03	0.80-1.32
2 or more	36	8.4	168	9.9		0.84	0.53-1.31
Multi-fetal pregnancy					0.794		
No	408	95.3	1629	95.6		reference	
Yes	20	4.7	75	4.4		1.07	0.65-1.77
Marital status					0.934		
No	51	11.9	208	12.2		reference	
Yes	377	88.1	1496	87.8		0.93	0.63-1.38
Excessive pregnant weight gain^b^					<0.001***		
No	85	19.9	1294	75.9		reference	
Yes	343	80.1	410	24.1		14.50***	11.02-19.08
Smoking					0.012*		
No	342	79.9	1448	85.0		reference	
Yes	86	20.1	256	15.0		1.79***	1.35-2.38
Delivery type					0.511		
Vaginal delivery	256	59.8	987	57.9		reference	
Cesarean delivery	172	40.2	717	42.1		0.94	0.75-1.17
Severity of GH-PE					<0.001***		
GH alone	257	60.0	681	40.0		reference	
PE	171	40.0	1023	60.0		2.46***	1.97-3.07
Co-morbidities							
Cardiovascular disease	10	2.3	36	2.1	0.713	1.12	0.56-2.26
Respiratory tract disease	12	2.8	43	2.5	0.734	1.12	0.59-2.14
Overt diabetes mellitus	86	20.1	168	9.9	<0.001***	2.30***	1.73-3.06
Gestational diabetes mellitus	257	60.0	330	19.4	<0.001***	6.25***	4.98-7.85
Thyroid disease	21	5.0	85	5.0	0.976	1.02	0.63-1.66
Renal disease	22	5.3	84	4.9	0.802	1.08	0.67-1.75

Data are expressed as the number (%) or mean ± standard deviation (SD), as appropriate *p<0.05, **p<0.01, ***p<0.01, by chi-square test, Student's t-test, or logistic regression analysis, as appropriate ^a^ Logistic regression analysis of adjusted odds ratio (OR) and 95% confidence interval (CI) for the occurrence of CHTN, compared with the reference (NBP) group ^b^ Weight gain ≥10 kgw during pregnancy, measured at 28 weeks' gestation
